# Spesolimab for acute-flare generalized pustular psoriasis with severe erythema and plaques: A case report

**DOI:** 10.1097/MD.0000000000044739

**Published:** 2025-10-24

**Authors:** Keyi Yu, Cheng Cao, Xingang Wu

**Affiliations:** a Department of Dermatology, Hangzhou Third Hospital Affiliated to Zhejiang Chinese Medical University, Hangzhou, Zhejiang Province, China; b Department of Dermatology, Hangzhou Third People’s Hospital, Hangzhou, Zhejiang Province, China.

**Keywords:** acute flare, biologics, IL-36 inhibitor, pustular psoriasis, spesolimab

## Abstract

**Rationale:**

Psoriasis is a prevalent, chronic papulosquamous skin disease, with generalized pustular psoriasis (GPP) representing a severe variant. Dysregulation of the IL-36 signaling pathway plays a crucial role in GPP, making biologics targeting this pathway the current therapeutic focus.

**Patient concerns:**

An elderly male patient presented with recurrent generalized erythematous, scaly patches and pustules. His medical history included GPP, psoriatic arthritis, rheumatoid arthritis, and type 2 diabetes mellitus. Previous treatments included methotrexate, glimepiride, metformin and voglibose, and adalimumab.

**Diagnoses:**

GPP was diagnosed based on clinical presentation, laboratory findings, and histopathological examination.

**Interventions:**

Due to inadequate response to previous treatments, spesolimab (900 mg) was administered via intravenous infusion.

**Outcomes:**

Skin lesions subsided rapidly within 1 week after the initial dose. At the 6-month follow-up, the patient remained in complete clinical remission without adverse events.

**Lessons:**

This case demonstrates that spesolimab is highly effective not only for pustules in GPP but also promotes excellent regression of erythema and plaques.

## 1. Introduction

Psoriasis is a prevalent, chronic papulosquamous skin disease affecting over 125 million individuals worldwide. It manifests as sporadic or diffuse scaly erythematous plaques.^[1]^ Plaque psoriasis, the most common subtype, has prompted a paradigm shift in treatment guidelines for moderate-to-severe cases. Current strategies prioritize combining biologics with oral medications or phototherapy over traditional step therapy.^[2]^ First-line therapies such as methotrexate, oral retinoic acid, and cyclosporin are commonly utilized but often limited by contraindications and suboptimal efficacy. Widely utilized biologics include TNF-α inhibitors (infliximab and adalimumab), IL-17 inhibitors (secukinumab and ixekizumab), and IL-12/23 inhibitors (ustekinumab). Nevertheless, data on spesolimab’s efficacy in plaque psoriasis remain limited.^[3]^

In generalized pustular psoriasis (GPP), dysregulation of the IL-36 signaling pathway plays a crucial role. Spesolimab, an IL-36 receptor (IL-36R) inhibitor, blocks IL-36R activation and has demonstrated significant efficacy in treating pustular psoriasis,^[4]^ establishing it as a potent therapeutic option. Here, we present a case of GPP along with severe erythema and plaques that showed substantial improvement following spesolimab treatment.

## 2. Case report

A 70-year-old male patient presented to our hospital with a recurrence of erythematous scales and pustules involving > 90% body surface area, having relapsed 4 days prior. He had a 25-year history of this condition, previously diagnosed as “erythrodermic psoriasis” and managed at our institution 1 year earlier. His medical history included GPP, psoriatic arthritis, rheumatoid arthritis, and type 2 diabetes. Previous treatments included methotrexate, glimepiride, metformin, and voglibose. Following clinical improvement, he was discharged on adalimumab as maintenance therapy. The patient received nearly 48 weeks of adalimumab 1 year ago: an initial 80 mg subcutaneous injection, followed by maintenance with 40 mg subcutaneously every 2 weeks starting 1 week later. Although skin lesions demonstrated significant improvement after 48 weeks, maintenance therapy was discontinued. Three months after discontinuing adalimumab, the patient developed progressive deterioration of cutaneous manifestations. He then reinitiated adalimumab, receiving two 80 mg subcutaneous doses at 2-week intervals. The rash showed no clinical resolution, prompting therapy discontinuation due to inadequate response. One month after cessation, he developed recurrent widespread erythematous plaques, pustules, and yellow crusting, accompanied by generalized pain and pruritus.

**Figure 1.**
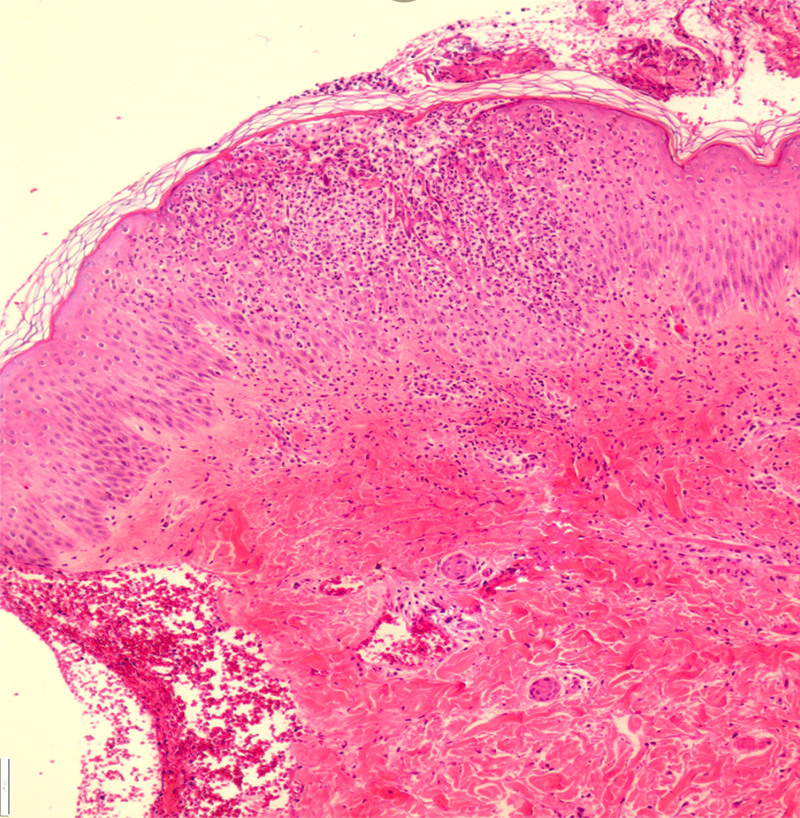
Histopathologic analysis of the skin infiltrate. Significant neutrophil infiltration in the epidermis and cuticle layer, stratum spinosum hypertrophy, hyperextension of the dermal protuberance, and infiltration of neutrophils and lymphocytes in the superficial dermal blood vessels.

Upon admission, the patient exhibited fever (38°C) with elevated inflammatory markers: white blood cell 12.9 × 10^9^/L, C-reactive protein 280 mg/L, erythrocyte sedimentation rate 66 mm/h, and procalcitonin 0.27 ng/mL (Table 1). Histopathological examination confirmed pustular psoriasis (Fig. 1). The diagnosis of GPP was established, with severe impact on quality of life (dermatology life quality index = 35), high disease activity (generalized pustular psoriasis area and severity index [GPPASI] = 45.6, generalized pustular psoriasis physician global assessment [GPPGA] = 3.5), and severe skin pain (numerical rating scale for pain = 8/10). Concomitant plaque psoriasis on the trunk and lower limbs yielded a psoriasis area and severity index score of 12.

**Table 1:** Selected laboratory indicators in the patient before and after spesolimab treatment.

Indicators (normal range)	D1	D7	D14	2 mo	4 mo	6 mo
WBC, 10^9^/L (3.5–9.5)	16.3	10.2	6.3	5.2	7.4	6.1
CRP, mg/L (<10)	280	15	9	5	6	3
ESR, mm/h (<20)	66	22	18	12	15	11
PCT, ng/mL (<0.05)	0.27	0.05	0.02	/	/	/
Creatinine, μmol/L (57–97)	53	67	71	84	78	69
ALT, U/L (0–50)	24	28	22	26	31	34
AST, U/L (15–40)	15	19	23	21	23	26
Sodium, mmol/L (135–145)	137	142	138	/	/	/
Potassium, mmol/L (3.50–5.30)	3.15	3.60	4.24	/	/	/
Serum albumin, g/L (40.0–55.0)	25.9	38.7	46.3	/	/	/

ALT = alanine transaminase, AST = aspartate transaminase, CRP = C-reactive protein, ESR = erythrocyte sedimentation rate, PCT = procalcitonin, WBC = white blood cell.

**Figure 2.**
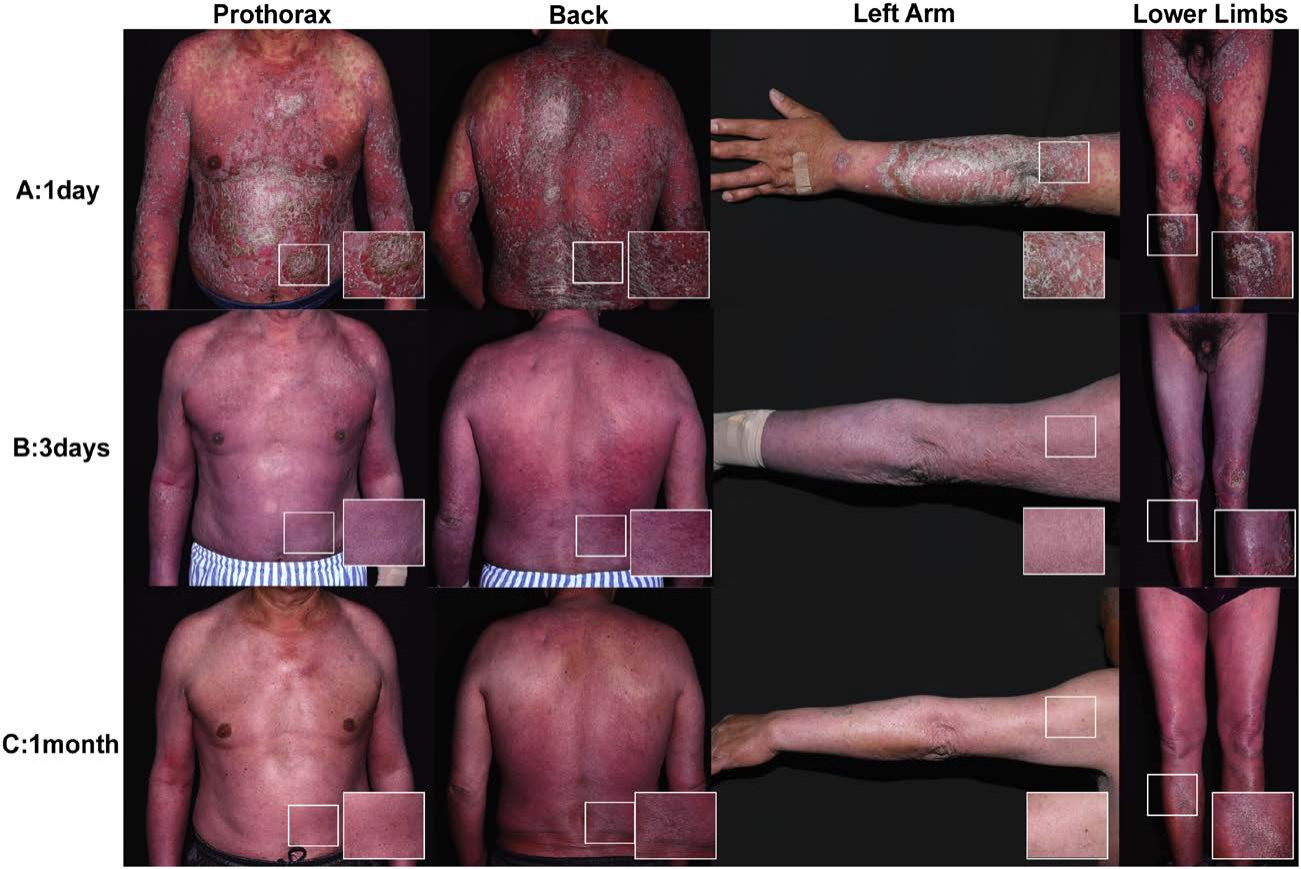
Clinical course of desquamation of erythema pustules in the patient. (A) Generalized erythematous patches with pustules and areas of desquamation on the initial presentation (GPPASI: 45.6; GPPGA: 3.5; PASI: 12). (B) Three days after the first dose of spesolimab 900 mg (GPPASI: 1.8; GPPGA: 1; PASI: 2). (C) One month after the first dose of spesolimab 900 mg (GPPASI: 0; GPPGA: 0; PASI: 0). GPPASI = generalized pustular psoriasis area and severity index, GPPGA = generalized pustular psoriasis physician global assessment, PASI = psoriasis area and severity index.

Following pharmacokinetic and clinical trial protocols, the patient received a single intravenous infusion of spesolimab 900 mg (dose calculated for 76 kg body weight).^[4,5]^ Remarkable clinical improvement was observed on day 7 post-infusion, including rapid and near-complete lesion clearance (GPPASI = 0.5; pustulation sub-score = 0; GPPGA = 0; psoriasis area and severity index = 1) (Fig. 2). Furthermore, his pruritus and pain symptoms improved significantly, accompanied by a substantial enhancement in his quality of life (numerical rating scale for pain = 0/10; dermatology life quality index = 1). Inflammatory markers demonstrated marked improvement from baseline, normalizing within 14 days (Table 1).

Given the patient’s significant symptomatic improvement and sustained clinical stability, no additional doses of spesolimab were administered. Regular follow-up was implemented, with the patient maintaining complete clinical remission for both GPP and plaque psoriasis at the 6-month assessment. Liver function parameters remained within normal limits, and no infectious complications were observed (Table 1), confirming the safety of spesolimab in this case.

## 3. Discussion

Psoriasis is an immune-mediated inflammatory dermatosis characterized by epidermal hyperproliferation, aberrant keratinocyte differentiation, and dysregulated local inflammatory mediators.^[6]^ GPP manifests as dense superficial aseptic pustules on psoriasis lesions, potentially accompanied by systemic manifestations including fever, hypoproteinemia, hypocalcemia, and hepatic or renal dysfunction. The condition exhibits a highly variable clinical course with relapse–remission characteristics, where severe symptoms and complications such as sepsis, neutrophilic cholangitis, heart failure, and renal failure often necessitate emergency intervention.^[7]^

Although GPP and plaque psoriasis may coexist, they demonstrate distinct clinical, histopathological, and genetic underpinnings. Consequently, GPP should be recognized as a distinct entity, though its management often aligns with psoriasis treatment paradigms.^[8]^ However, limited data exist regarding the efficacy of biologics approved for plaque psoriasis, and their use frequently proves insufficient for GPP management.^[8]^

Interleukin-36 plays a pivotal role in GPP pathogenesis by driving neutrophils and Th17 cells’ recruitment and activation within psoriatic lesions.^[9]^ Spesolimab inhibits IL-36R, disrupting the inflammatory cascade mediated by IL-36α/γ cytokines. These cytokines induce pro-inflammatory mediators in psoriatic plaques, disrupt epidermal differentiation, and promote pathological angiogenesis. This inhibition normalizes keratinocyte function by restoring IL-36-impaired differentiation processes, as IL-36R blockade rescues physiological levels of differentiation markers. It also reduces vascular inflammation by suppressing IL-36-induced endothelial cell activation, including decreased proliferation, tube formation, and expression of adhesion molecules such as intracellular adhesion molecule-1 and vascular cell adhesion molecule, which otherwise drive leukocyte recruitment. Collectively, these actions resolve both pustules and associated plaques by targeting IL-36R-dependent pathways specific to plaque pathogenesis.^[10]^

This novel IL-36R inhibitor has demonstrated clinical efficacy in pustular psoriasis. A recent case report documented a patient receiving two 900 mg IV spesolimab doses (administered 1 week apart) who achieved marked improvement within 7 days, complete remission by week 2, and sustained remission at 12 months with minimal plaques. This patient had no complex comorbidities.^[11]^ In contrast, our 70-year-old patient with multiple comorbidities (psoriatic arthritis, rheumatoid arthritis, type 2 diabetes) achieved similarly rapid and sustained remission with a single 900 mg IV dose. This highlights spesolimab’s efficacy across dosing regimens and patient populations, supporting its utility in complex medical histories. Notably, the single-dose approach simplifies treatment logistics, particularly for patients with multiple comorbidities. It also reduces direct drug costs compared to multi-dose regimens and minimizes hospitalization needs by enabling rapid symptom control.

Potestio et al^[12]^ summarized existing literature on spesolimab for GPP. While clinical trials and real-world experience confirm its effectiveness and safety, long-term data remain limited. Our case demonstrated that short-term single-dose spesolimab effectively promotes plaque regression, controls disease progression, and provides substantial symptomatic improvement without adverse events. Its favorable administration profile and minimal adverse effects establish spesolimab as a beneficial treatment option.

However, the long-term efficacy of the single-dose regimen remains uncertain due to limited follow-up data beyond short-to-moderate durations. Larger clinical studies and longer-term observation will be necessary to clarify the durability of responses with this dosing strategy.

## Author contributions

**Writing – original draft:** Keyi Yu, Cheng Cao.

**Writing – review & editing:** Xingang Wu.
